# Attachment Style, Task Difficulty, and Feedback Type: Effects on Cognitive Load

**DOI:** 10.3390/bs15040427

**Published:** 2025-03-27

**Authors:** Dor Mizrahi, Ilan Laufer, Inon Zuckerman

**Affiliations:** Department of Industrial Engineering and Management, Ariel University, Ariel 4070000, Israel; ilanl@ariel.ac.il (I.L.); inonzu@ariel.ac.il (I.Z.)

**Keywords:** attachment style, cognitive load, EEG, theta/beta ratio, task difficulty, feedback, inhibitory control, emotional regulation, human–agent interaction

## Abstract

This study examines how attachment styles influence cognitive load during a dot counting task with varying difficulty levels and feedback types. From an initial pool of 96 participants, 27 were selected based on attachment classifications from the Experiences in Close Relationships-Revised (ECR-R) questionnaire. These participants completed the task while receiving personal and group-based feedback, and EEG recordings monitored cognitive load using the theta/beta ratio (TBR). Results show that negative personal feedback consistently elevated cognitive load across all attachment styles. Avoidant and fearful-avoidant individuals did not exhibit significant differences in cognitive load between positive and negative group feedback, suggesting a relatively stable response regardless of feedback valence. In contrast, securely attached individuals showed increased cognitive load under negative feedback conditions. Furthermore, individuals with higher attachment-related anxiety or avoidance experienced more pronounced increases in cognitive load as task difficulty increased. These findings suggest that attachment-related differences shape cognitive responses to feedback and task complexity level. The study highlights the role of adaptive feedback strategies in optimizing cognitive engagement across different attachment profiles.

## 1. Introduction

The way individuals manage their mental resources has a profound impact on their ability to perform tasks, especially when faced with challenges such as increasing task complexity or varying feedback. Cognitive load, which refers to the mental effort involved in processing information, solving problems, and making decisions, is shaped by individual differences, including attachment styles (Comte et al., 2024; Peng et al., 2024; Zuckerman et al., 2023).

Bowlby’s attachment theory (Ogle & Cozza, 2017; van der Horst et al., 2024) provides a foundational framework for understanding how early emotional bonds with caregivers shape psychological and behavioral patterns throughout life. Attachment styles can be classified into secure, anxious, avoidant, and fearful-avoidant categories. Each reflects a unique way of responding to stress, regulating emotions, and managing relationships. While much of the research explored how attachment influences social and emotional behavior, there is growing interest in its role in cognitive processes, particularly under stress or environmental demands (Comte et al., 2024; Cushing et al., 2024; Lu et al., 2024; Peng et al., 2024).

Despite this interest, the connection between attachment styles and cognitive load remains relatively unexplored. Given that attachment styles influence emotional regulation and attentional control, they likely play a crucial role in how individuals allocate cognitive resources during difficult tasks (Gillath et al., 2009; Karreman & Vingerhoets, 2019; Warren et al., 2010). Insecure attachment styles, in particular, are associated with heightened sensitivity to stress and feedback, which can impact cognitive efficiency (Bendezú et al., 2019; Warren et al., 2010). Investigating these relationships is essential for understanding how emotional and cognitive regulation interact and how adaptive feedback strategies can be developed to optimize cognitive performance across different attachment profiles (Bar-Haim et al., 2007; Gillath et al., 2005; Stout et al., 2017). Examining how attachment styles interact with cognitive load can help identify the underlying mechanisms that shape cognitive and emotional regulation. This study aims to bridge this gap by investigating how attachment-related emotional regulation influences cognitive effort in response to varying task difficulties and feedback conditions. Specifically, we distinguish between two types of feedback: individual (personalized) feedback, which reflects the participant’s own performance, and group feedback, which is based on the collective performance of the group and introduces a social comparison component. By examining whether individuals with different attachment styles allocate cognitive resources differently under these conditions, and whether task difficulty further modulates these effects, this study provides insight into the role of emotional regulation in feedback processing and its implications for cognitive performance.

### 1.1. Attachment Styles and Cognitive Resource Allocation

Attachment styles are closely linked to emotional regulation, which affects an individual’s ability to maintain focus, control impulses, and allocate mental effort to complex tasks (Gillath et al., 2009; Karreman & Vingerhoets, 2019; Warren et al., 2010). People with secure attachment generally manage cognitive resources effectively, even in stressful situations or with emotional distractors (Bendezú et al., 2019; Warren et al., 2010). In contrast, those with anxious or avoidant attachment often face challenges. For instance, anxious individuals may become overly focused on potential threats, which consumes cognitive resources (Bar-Haim et al., 2007; Stout et al., 2017). Avoidant individuals, on the other hand, may initially suppress emotional responses, but they often fail to maintain this suppression when faced with a cognitive load, potentially impairing their ability to concentrate on difficult tasks (Gillath et al., 2005).

Cognitive load theory (CLT) categorizes cognitive load into intrinsic, extraneous, and germane types (Orru & Longo, 2019). Intrinsic load depends on task complexity, influenced by the number of interacting elements that must be processed simultaneously (element interactivity) and an individual’s prior knowledge (Seufert et al., 2007). Extraneous load arises from inefficient task design (Lange et al., 2017), while germane load reflects mental effort dedicated to learning and adaptation (Stiller & Schworm, 2019). This study focuses on intrinsic and germane load, as task difficulty affects intrinsic load by increasing cognitive effort for visual comparisons and judgments. Germane load is examined in relation to feedback processing and cognitive resource allocation across attachment styles.

Neurophysiological measures, particularly electroencephalography (EEG), have been widely used to assess cognitive load in response to task complexity. EEG-based cognitive load prediction typically relied on spectral signatures and functional connectivity, but recent research highlights the importance of integrating spatial, temporal, and spectral EEG features to improve multilevel cognitive load assessment (Liu et al., 2023; Suzuki et al., 2024). Studies demonstrated that increased cognitive load is associated with elevated theta power, reduced alpha power, and significant changes in interchannel connectivity and microstates, suggesting that EEG provides a multidimensional representation of cognitive effort (Liu et al., 2023). Power spectral analyses, in particular, have been instrumental in capturing real-time variations in cognitive effort allocation (Suzuki et al., 2024). Among these, the theta/beta ratio (TBR) has been explored as a potential indicator of cognitive effort, particularly in relation to attentional control and executive function (Angelidis et al., 2016, 2018; Putman et al., 2014; D.-W. Zhang et al., 2019). Lower TBR is associated with enhanced executive and attentional control, as well as effective regulation of attentional bias toward emotionally threatening stimuli, making it a potential biomarker for cognitive and emotional regulation (Angelidis et al., 2016, 2018). By integrating CLT with attachment-related emotional regulation, this study explores how feedback and task difficulty shape cognitive effort across different attachment styles (Edelstein, 2006).

Insecure attachment styles further amplify cognitive demands due to heightened sensitivity to stress and emotional feedback. Anxious attachment is associated with faster threat detection and schema-driven “sentinel” processing, while avoidant attachment engages a “rapid fight–flight schema,” often relying on suppression strategies that impair task performance under stress. Neurophysiological studies show increased prefrontal activity and reduced attentional allocation (e.g., lower P3 amplitudes) in insecurely attached individuals during emotional tasks, underscoring the cognitive costs of less efficient emotion regulation strategies (Ein-Dor et al., 2011; Leyh et al., 2016; Warren et al., 2010).

### 1.2. Feedback, Emotional Regulation, and Inhibitory Control

Emotional regulation and inhibitory control are important factors through which attachment style influences cognitive functioning. These processes determine how individuals process feedback, regulate emotions, and manage cognitive demands, shaping their ability to adapt and perform effectively.

Emotional regulation governs responses to feedback, influencing whether it enhances or disrupts cognitive functioning. Securely attached individuals integrate both positive and negative feedback constructively (Ein-Dor et al., 2011). Anxiously attached individuals ruminate on negative feedback while disregarding positive reinforcement, increasing cognitive strain (Gentzler et al., 2010; Zuckerman et al., 2023). Avoidantly attached individuals suppress emotional responses, which may initially reduce stress, but can hinder engagement with feedback over time (Ran & Zhang, 2018).

Feedback further interacts with these regulatory patterns. Securely attached individuals use reinforcement and constructive criticism adaptively (Ein-Dor et al., 2011). Avoidantly attached individuals may disengage from socially evaluative feedback, limiting their responsiveness to both positive and negative cues (Gentzler et al., 2010). Anxiously attached individuals focus excessively on negative feedback, increasing emotional distress and making it harder to adjust behavior (Gentzler et al., 2010; Zuckerman et al., 2023).

Inhibitory control plays an important role in managing cognitive resources by filtering distractions and regulating attentional focus. Securely attached individuals demonstrate stronger inhibitory control, helping them stay engaged and process feedback adaptively (Kim et al., 2020; Zuckerman et al., 2023). In contrast, individuals with attachment-related anxiety may struggle with inhibitory control due to heightened emotional reactivity, making it harder to regulate cognitive effort, especially when faced with negative feedback (Gentzler et al., 2010). Avoidantly attached individuals may suppress emotional responses but still encounter challenges in maintaining cognitive flexibility in complex tasks (Ran & Zhang, 2018).

Cognitive functioning emerges from the interplay of these processes. Emotional regulation determines whether feedback is integrated constructively or becomes a source of cognitive strain, while inhibitory control helps allocate cognitive resources efficiently. Secure attachment fosters emotional stability and attentional control, promoting adaptability in cognitive tasks (Zuckerman et al., 2023). In contrast, insecure attachment, whether through heightened emotional sensitivity or disengagement, can lead to cognitive overload and inflexible problem solving (Gentzler et al., 2010; Ran & Zhang, 2018). Understanding these relationships clarifies how attachment styles shape cognitive performance and provides a foundation for strategies that support emotional resilience and adaptability.

Neurobiologically, these patterns are reflected in brain activity. Securely attached individuals show balanced activation in reward- and threat-related regions, such as the ventral striatum and amygdala, supporting emotional stability (Vrtička et al., 2008). Avoidantly attached individuals exhibit weaker striatal responses to positive feedback, reducing motivation and engagement (Ran & Zhang, 2018). Anxiously attached individuals display heightened amygdala activation in response to negative feedback, reinforcing rumination and stress sensitivity (Gentzler et al., 2010; Vrtička et al., 2008).

### 1.3. Objectives of the Current Study

#### 1.3.1. Theoretical Background and Rationale

Building on insights from attachment theory and cognitive load research, this study explores how attachment styles influence cognitive performance under varying task complexities and feedback conditions. Attachment styles are linked to emotional regulation and cognitive biases (Ein-Dor et al., 2011; Leyh et al., 2016), yet their effect on real-time cognitive load in response to feedback remains unclear. Prior research examined biased threat processing and attention allocation in insecure attachment, particularly in emotional contexts (Leyh et al., 2016), but has not systematically examined how attachment moderates cognitive effort under varying task difficulties.

Secure individuals process social information flexibly, while insecure individuals either avoid distressing stimuli or engage with a negative bias (Dykas & Cassidy, 2011). These biases extend to cognitive engagement, where avoidant individuals seek cognitive closure and limit information processing, while anxious individuals struggle with distraction and adaptation (Mikulincer, 1997). However, most studies focused on emotional stimuli rather than feedback-driven cognitive engagement.

#### 1.3.2. Cognitive Load and Feedback Processing

While neuroscience research, including EEG-based studies (Buchheim et al., 2017), examined attachment-related differences in emotional reactivity, inhibitory control, and attentional biases, prior studies primarily investigated how individuals suppress responses to emotional or threatening stimuli, rather than how they regulate cognitive effort allocation in feedback-based tasks. Event-related potentials (e.g., P300) have been used to measure attentional biases in response to emotional stimuli (Leyh et al., 2016), but these approaches do not capture how cognitive resources are adjusted throughout task performance in response to feedback and task difficulty fluctuations.

Inhibitory control has been explored in attachment research (Buchheim et al., 2017), primarily through response inhibition paradigms (e.g., Go/No-Go tasks) measuring suppression of motor responses, rather than in studies assessing how cognitive effort is actively sustained or modulated in response to evaluative feedback. Unlike previous studies that focused on momentary attentional shifts or inhibitory control in response inhibition tasks (Buchheim et al., 2017), our study investigates how individuals actively regulate cognitive effort in response to varying task demands. By using the theta/beta ratio (TBR) as a neurophysiological marker, we provide a continuous measure of cognitive effort, offering a distinct approach from prior studies focusing primarily on emotional reactivity and inhibitory control.

#### 1.3.3. Methodological Approach and Novel Contribution

Cognitive load can be assessed using behavioral (e.g., reaction time, error rates), physiological (e.g., heart rate variability, pupil dilation, and blink rate), and EEG-based measures (e.g., spectral power ratios, P300). While physiological indices such as pupil diameter change, heart rate, and blink rate provide insight into cognitive load, they often require trend analysis across multiple parameters and can be influenced by external factors such as emotional state and environmental conditions (Ahmad et al., 2020). Self-reported measures, while widely used for their ease of implementation and validity, are retrospective and may not capture real-time cognitive fluctuations. EEG-based methods provide more direct neural markers of cognitive effort. Event-related potentials such as P300 capture transient attentional shifts but do not reflect sustained cognitive effort regulation over time (Angelidis et al., 2016).

We selected TBR as a neurophysiological marker because it provides a stable and objective measure of sustained cognitive effort. Unlike P300, which reflects brief attentional shifts, TBR tracks ongoing cognitive resource allocation, making it well-suited for studying feedback-driven cognitive regulation across different task difficulty levels (Angelidis et al., 2016; Laufer et al., 2022). TBR is also linked to attentional control and cognitive engagement, making it particularly relevant for examining how attachment-related emotional regulation influences cognitive effort (Suzuki et al., 2024).

This study offers a novel contribution by integrating attachment theory with EEG-based cognitive load assessment, specifically through the use of theta/beta ratio (TBR) as a biomarker for cognitive effort. While previous studies examined emotional regulation and inhibitory control in attachment styles, our approach uniquely examines how attachment influences real-time cognitive engagement in response to both task difficulty and feedback conditions. By distinguishing between personal and group feedback and analyzing their differential impacts on cognitive load across attachment profiles, this study advances the understanding of feedback processing and emotional regulation in task performance.

#### 1.3.4. Limitations and Hypothesis Development

Despite its advantages, TBR has limitations. It lacks specificity in distinguishing between cognitive disorders such as ADHD and learning disabilities (Nezhad et al., 2025). Additionally, TBR may not always differentiate between cognitive effort driven by task difficulty and effort influenced by individual problem-solving strategies, such as intuitive versus deliberate thinking (Laufer et al., 2022). However, despite these limitations, TBR remains a well-established neurophysiological marker of cognitive effort and attentional control, particularly in structured task environments where cognitive load fluctuates in response to feedback and task difficulty. Unlike behavioral or self-reported measures, TBR provides an objective, continuous index of cognitive effort, making it well-suited for examining how attachment-related emotional regulation influences cognitive engagement in real time.

Using a dot counting task (Vrtička et al., 2008) designed to manipulate both task difficulty and feedback type, we examine the interaction between attachment-related emotional regulation and cognitive effort. Attachment styles were assessed using the Experiences in Close Relationships-Revised (ECR-R) questionnaire (Sibley & Liu, 2004), while EEG measured changes in theta/beta ratio (TBR), a reliable biomarker of executive attentional control with strong test–retest reliability and predictive validity for cognitive performance (Angelidis et al., 2016).

We hypothesize that (1) individuals with anxious attachment styles will react more strongly to negative feedback than to positive feedback, as well as more strongly than securely attached individuals. Their heightened sensitivity to perceived social punishment, combined with a tendency to ruminate on negative experiences, is likely to increase their cognitive load (Stanton & Campbell, 2015; Vrtička et al., 2008). This effect may be particularly pronounced during difficult tasks, as their focus shifts toward processing potential threats rather than the task itself. In contrast, we hypothesize that (2) individuals with avoidant attachment styles will be less responsive to positive feedback compared to securely attached individuals. Their reliance on suppression strategies to manage stress suggests that they may downplay the significance of affirming feedback, leading to a diminished cognitive response.

As task demands rise, avoidant individuals may struggle to sustain their regulatory strategies, potentially leading to heightened cognitive strain. This aligns with findings showing that while avoidant attachment is linked to better performance on attention tasks unrelated to attachment, reminders of attachment insecurity can hinder their performance (Gillath et al., 2009).

## 2. Materials and Methods

### 2.1. Description of the Dot Counting Task

**Figure 1:**
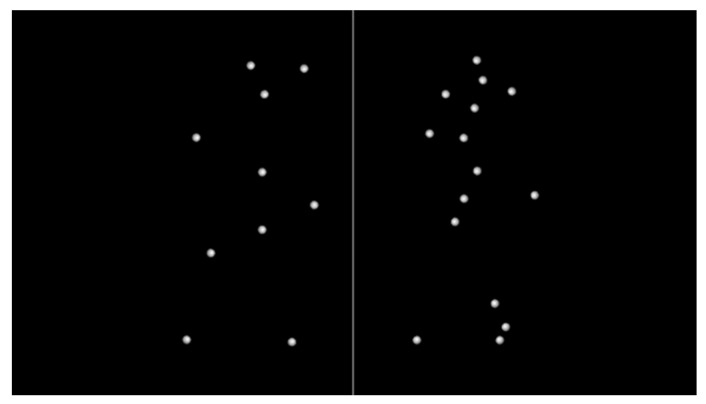
Visualization of the dot counting task screen, showing the arrangement of white dots on either side of a vertical divider. The example illustrates a difficulty level of 5, with 10 dots on the left side and 15 dots on the right side.

The dot counting task (Vrtička et al., 2008) was designed to evaluate cognitive effort under varying levels of difficulty. Each trial began with a fixation screen displaying a white cross on a black background for a randomly assigned duration of one to three seconds, allowing participants to focus their attention. This was followed by a screen divided into two halves by a vertical white line, with white dots randomly distributed on either side. Participants were shown this configuration for 500 milliseconds, after which the screen disappeared. They were then given up to one second to determine which side contained more dots and to indicate their choice by pressing the corresponding left or right arrow key. The difficulty of each trial depended on the difference in the number of dots between the two sides, ranging from 0 (the most challenging) to 5 (the easiest). The number of dots on each side varied between 10 and 15, ensuring that difficulty was primarily influenced by relative differences rather than the absolute number of dots. To ensure a fair assessment of performance, the difficulty levels were evenly and randomly distributed across the trials. Figure 1 displays an example of the dot counting task at a difficulty level of 5, where the left side contains 10 dots and the right side contains 15 dots.

After making their selection, participants received feedback displayed on a screen for one second. This feedback included both individual and group-based components. Individual feedback informed participants whether their response was correct or incorrect, conveyed through written text stating either “You won!” for correct answers or “You lost!” for incorrect answers. Group feedback compared the participant’s response speed to the group average, with a happy smiley indicating faster-than-average performance and a sad smiley indicating slower-than-average performance. Monetary rewards ranging from NIS 100 to NIS 150 were offered based on performance, incentivizing consistent effort throughout the task.

**Figure 2:**
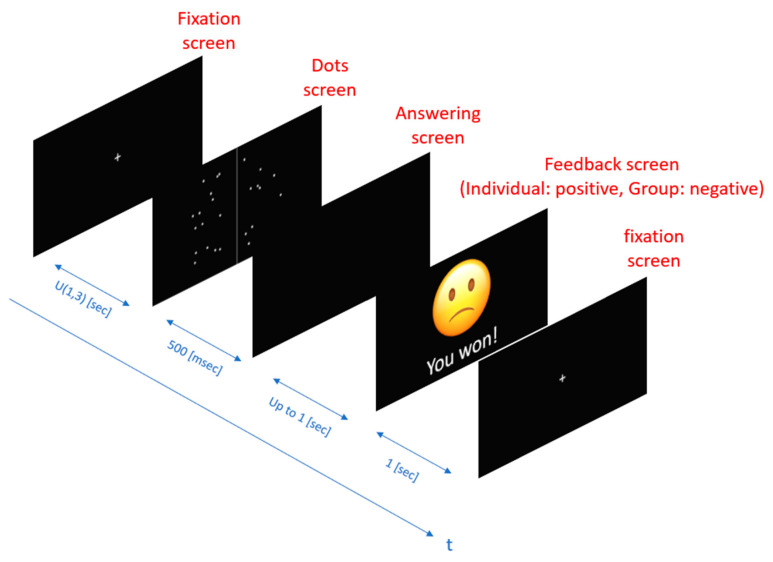
Sequence of the dot counting task, illustrating each stage (fixation screen, dots screen, answering screen, and feedback screen) with their respective display durations.

The sequence of screens within a single trial is illustrated in Figure 2, which depicts the progression from the fixation screen to the dots screen, followed by the answering phase and the feedback phase, along with their respective display durations. This structured setup ensured consistency across trials and enabled the systematic manipulation of task difficulty and feedback conditions to assess cognitive performance under varying levels of challenge (Archer-Kath et al., 1994; Tindale et al., 1991; Vrtička et al., 2008).

### 2.2. Study Population and Initial Assessment

Participants were recruited from a pool of 96 fourth-year engineering students, aged 20 to 35 years (mean = 24.25, SD = 2.07), including 46 females. All participants were right-handed and reported no history of neurological conditions. Attachment styles were assessed using the Experiences in Close Relationships-Revised (ECR-R) questionnaire (Sibley et al., 2005; Sibley & Liu, 2004), a validated self-report instrument that evaluates adult attachment on two dimensions: anxiety and avoidance. Scores range from 1 to 7, with lower scores indicating secure attachment, and higher scores in both dimensions associated with fearful-avoidant or disorganized attachment styles. Responses to the ECR-R questionnaire were analyzed using a k-means clustering algorithm (Jain, 2008), which identified natural groupings of attachment styles within the participant pool. The elbow method was employed to determine the optimal number of clusters, balancing within-cluster compactness with diminishing returns in homogeneity. This analysis revealed four clusters corresponding to secure, anxious, avoidant, and fearful-avoidant attachment styles, consistent with prior literature (Bowlby, 1969; Jain, 2008).

**Figure 3:**
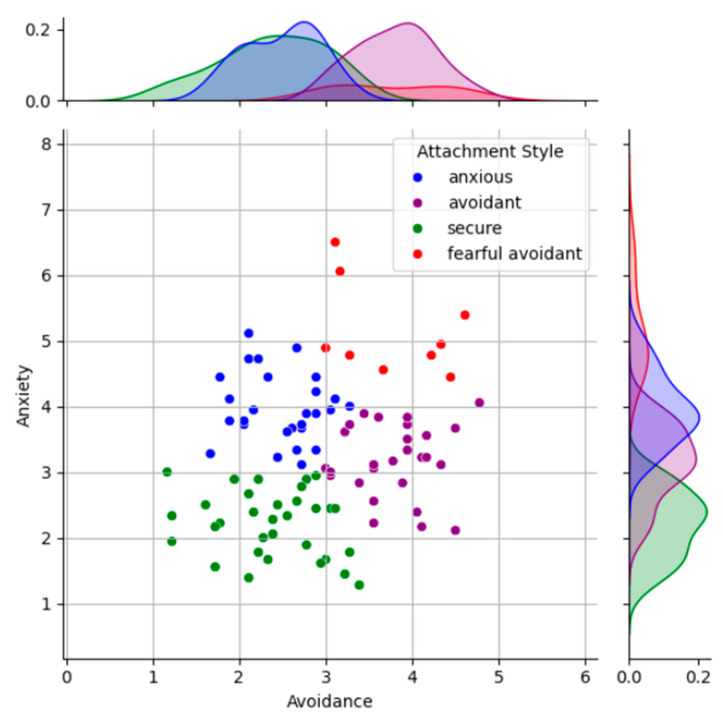
Attachment style classification derived from the ECR-R questionnaire using k-means clustering (K = 4), displaying the grouped attachment outcomes for the initial participant pool.

Figure 3 provides a representation of the clustering outcome, mapping participants’ anxiety and avoidance scores to their respective attachment categories. The scatter plot, generated through k-means clustering, categorizes participants into four distinct attachment groups: secure (green), anxious (blue), avoidant (purple), and fearful-avoidant (red). The accompanying density plots along the axes depict the distribution of anxiety and avoidance scores for each category. Secure participants are clustered in the lower left quadrant, characterized by low anxiety and avoidance scores, while fearful-avoidant participants occupy the upper right quadrant, marked by high scores in both dimensions. Anxiously attached individuals are positioned higher on the anxiety axis, and avoidant individuals are located further along the avoidance axis. The figure highlights the clear differentiation of attachment groups based on these two dimensions.

### 2.3. Sample Selection

From the identified clusters, 27 participants were selected using proportional allocation, ensuring that the sample accurately reflected the prevalence of attachment styles in the broader population. This approach involved selecting participants in proportion to the occurrence of each attachment style, resulting in a sample comprising 6 securely attached individuals and 21 with insecure attachment styles, distributed as 9 anxious, 7 avoidant, and 5 fearful-avoidant participants. After selection, these participants transitioned to the cognitive task phase, during which EEG recordings were conducted. By combining proportional allocation with k-means clustering, the sample distribution aligned with the four attachment groups widely recognized in the literature (Magai et al., 2001). This method preserved naturally occurring attachment style distributions, ensuring ecological validity and enhancing the study’s relevance to real-world contexts. Although this approach inherently resulted in unequal group sizes, these variations mirror natural population patterns and were necessary to maintain a representative sample.

### 2.4. Experimental Procedure and EEG Recording

The experiment consisted of two blocks of the dot counting task, with each block containing 20 trials. Participants were instructed to identify as quickly and accurately as possible which side of the screen displayed more dots. During this cognitive task, brain activity was recorded using a 16-electrode EEG system (g.USBamp bio-signal amplifier, g.tec, Schiedlberg, Austria) positioned according to the international 10–20 system (Khazi et al., 2012). Electrode impedance was maintained below 5000 ohms to ensure high data quality. The EEG signals were processed and stored using the OpenVibe software (version 3.6.0) for both signal acquisition and real-time processing (Renard et al., 2010). The behavioral experiment was presented using a Java 17–based application, developed by Dr. Dor Mizrahi from the NeuroIS Lab, Faculty of Engineering, Ariel University. This integration of behavioral and neurophysiological data allowed for an in-depth examination of cognitive processes modulated by attachment styles.

## 3. Data Processing, Results, and Analysis

### 3.1. Data Processing

EEG data were pre-processed using a protocol consistent with established methodologies (Laufer et al., 2022; Zuckerman et al., 2022) in EEGLAB (Delorme & Makeig, 2004) (version 14.1.1b). A bandpass filter in the [1, 32] Hz range was applied to isolate the relevant frequency components required for calculating the theta/beta ratio (TBR), a commonly used measure of cognitive load. To reduce interference, a notch filter at 50 Hz was employed to minimize power grid noise. The data were re-referenced by averaging across all channels, enhancing signal clarity. Artifact removal was performed using independent component analysis (ICA) on the entire EEG dataset. ICA is a computational technique that separates multivariate signals into statistically independent components. In the context of EEG data, ICA is particularly effective in isolating artifacts, such as eye movements, muscle noise, or line noise, from neural activity. By separating these independent components, ICA enables a clearer analysis of brain signals, improving the accuracy of subsequent data interpretation (Hyvärinen & Oja, 2000; Pester & Ligges, 2018; Vigário et al., 2000). The cleaned signal was then segmented into one-second epochs, starting from the presentation of feedback, with baseline correction applied to each epoch.

Following artifact removal, the data were downsampled from 512 Hz to 64 Hz to streamline analysis while maintaining signal fidelity, as supported by the Nyquist theorem. This lower sampling rate was adequate for TBR calculations and ensured computational efficiency. The TBR was computed using six frontal and prefrontal EEG electrodes (Fp1, F7, Fp2, F8, F3, and F4). The TBR was first calculated separately for each electrode using a three-level discrete wavelet transform (DWT) to estimate the relative energy in the theta and beta frequency bands. The ratio of theta to beta energy provided the TBR, an approach validated in prior studies for its reliability and alignment with established EEG analysis methods (Mizrahi et al., 2024b). The final TBR value was obtained by averaging across all six electrodes to enhance signal robustness and reduce noise.

Statistical analyses were conducted using Python’s statsmodels library for factorial ANOVA and post hoc Tukey’s HSD tests, scipy.stats for regression analysis, and numpy.trapz for calculating the area under the graph.

### 3.2. Attachment Style and Feedback Effects on Cognitive Load

#### 3.2.1. Statistical Results

A factorial ANOVA was conducted with attachment style (secure, anxious, avoidant, and fearful-avoidant) as a between-subjects factor, and group feedback (positive, negative) and personal feedback (positive, negative) as within-subjects factors on the TBR. Significant main effects were observed for attachment class (F(3,1064) = 21.62, *p* < 0.001), group feedback (F(1,1064) = 3.85, *p* < 0.05), and personal feedback (F(1,1064) = 27.58, *p* < 0.001). Additionally, significant two-way interactions were found between attachment class and group feedback (F(3,1064) = 49.88, *p* < 0.001), as well as attachment class and personal feedback (F(3,1064) = 39.88, *p* < 0.001), suggesting that the effects of feedback depend on the attachment style of the participant. While a significant three-way interaction between attachment class, group feedback, and personal feedback (F(3,1064) = 49.88, *p* < 0.001) was also identified, the present study focused on the two-way interactions, attachment class × group feedback and attachment class × personal feedback, to directly address the study’s core hypotheses. This focus allowed for a detailed examination of how attachment styles independently moderate responses to group and personal feedback.

To further investigate these interactions, post hoc comparisons were conducted using Tukey’s post hoc test, which inherently adjusts for multiple comparisons using the Tukey–Kramer method. This approach ensures that the reported *p*-values account for multiple pairwise comparisons, reducing the likelihood of type I errors.

#### 3.2.2. Interaction Between Attachment Class and Group Feedback

**Figure 4:**
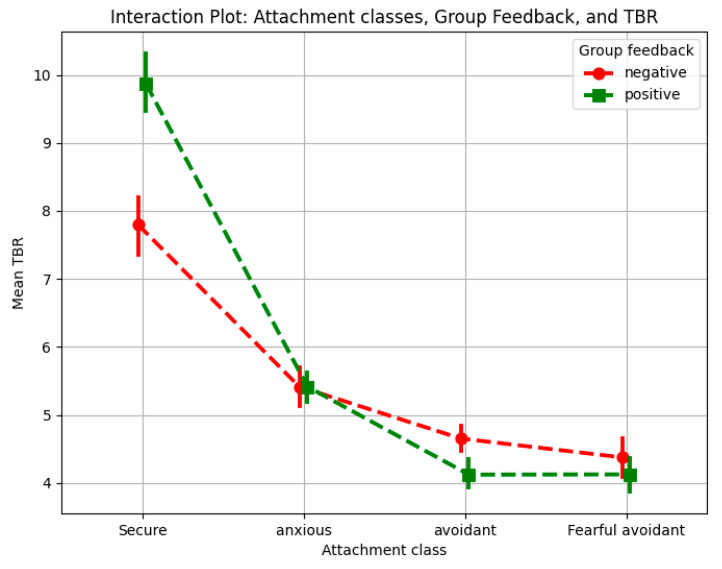
Interaction plot for attachment class and group feedback.

Figure 4 illustrates both a general separation between secure and insecure attachment groups in overall TBR levels and the interaction between attachment style and group feedback. Securely attached individuals exhibited lower overall cognitive load when processing feedback compared to insecurely attached individuals, aligning with theoretical models that suggest secure individuals process social information more efficiently through well-integrated emotional regulation mechanisms. Post hoc analyses revealed significant differences in TBR between feedback conditions across attachment groups. Securely attached individuals exhibited a significantly lower TBR under negative feedback compared to positive feedback (*p* < 0.05), indicating increased cognitive load when engaging with negative social evaluation. This aligns with research suggesting that secure individuals process both positive and negative social information openly and actively, rather than avoiding distressing feedback. Moreover, they tend to interpret negative feedback in a constructive manner, which may contribute to their increased cognitive effort when processing it (Dykas & Cassidy, 2011).

Anxiously attached individuals showed no significant TBR differences between positive and negative feedback (*p* < 0.05). This could reflect a tendency to filter out distressing information to minimize psychological discomfort, as suggested by prior research (Dykas & Cassidy, 2011). Their heightened sensitivity to social evaluation may lead them to engage with all feedback in a way that prevents excessive cognitive overload, rather than differentiating between positive and negative feedback (Y. Zhang et al., 2023).

fMRI research indicates that avoidant individuals are more efficient at disengaging from positive emotional stimuli but have difficulty disengaging from negative emotional stimuli. This suggests that positive feedback, which contradicts their habitual avoidance of social evaluation, may require additional cognitive resources to process, leading to an increase in cognitive load (Liu et al., 2017). Fearful-avoidant individuals showed a slight tendency toward lower TBR under positive feedback and higher TBR under negative feedback, though these differences were minimal and non-significant (*p* > 0.05), suggesting a weaker modulation in cognitive load by feedback valence compared to other attachment groups. Research on fear of negative evaluation suggests that individuals with heightened sensitivity to social judgment engage more deeply with negative feedback, increasing their cognitive load (Y. Zhang et al., 2023). This suggests that while positive feedback imposed cognitive demands, it did not elevate cognitive load to the same extent as it did for avoidant individuals, who may experience stronger cognitive conflict when confronted with affirming feedback.

These findings demonstrate an interaction between attachment style and feedback valence. Secure and anxiously attached individuals experienced greater cognitive load when receiving negative feedback, whereas avoidant and fearful-avoidant individuals exhibited higher cognitive load in response to positive feedback. This pattern aligns with prior research indicating that attachment-related emotional regulation influences how individuals allocate cognitive resources when processing social evaluation (Dykas & Cassidy, 2011).

#### 3.2.3. Interaction Between Attachment Class and Personal Feedback

**Figure 5:**
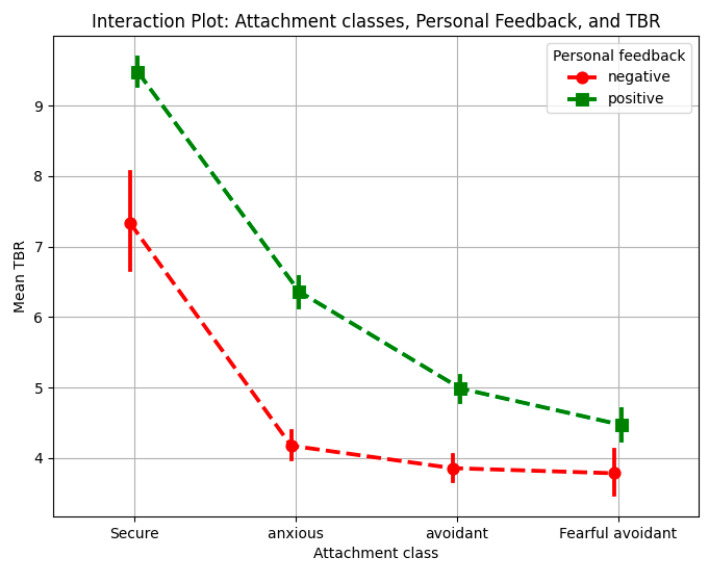
Interaction plot for attachment class and personal feedback.

The results presented in Figure 5 emphasize the consistent impact of personal feedback on cognitive load across all attachment styles, in contrast to the more variable patterns observed with group feedback (Figure 4). Personal feedback, conveyed through explicit textual messages (i.e., “You Won!” for positive feedback and “You Lost!” for negative feedback), consistently influenced TBR values across attachment profiles. Negative personal feedback was associated with lower TBR values compared to positive feedback, indicating higher cognitive load under conditions of negative feedback.

Securely attached individuals exhibited the largest difference in TBR between positive and negative feedback, suggesting that their capacity to process evaluative feedback deeply is accompanied by a stronger cognitive response to negative feedback. In contrast, individuals in the Fearful-Avoidant group showed the smallest difference, likely due to their heightened baseline stress levels and self-critical tendencies, which may dampen their responsiveness to feedback valence. Anxiously attached individuals demonstrated a moderate difference in TBR values between positive and negative feedback, reflecting their sensitivity to emotional feedback, particularly negative feedback. Avoidant individuals displayed a similar trend, though their reliance on emotional suppression strategies appeared to buffer the impact of feedback to some extent, resulting in slightly more differentiated cognitive responses than those seen in the fearful-avoidant group.

The results underline the consistent impact of personal feedback, with negative feedback increasing cognitive load across all attachment styles. In contrast, group feedback exhibited variability, with distinct patterns observed across attachment profiles. These findings emphasize the role of feedback type in shaping cognitive responses and suggest that tailoring feedback approaches to individual attachment styles could enhance emotional and cognitive outcomes in adaptive systems and learning environments.

### 3.3. Post Hoc Comparisons for Specific Feedback Conditions

To further elucidate the impact of feedback and attachment styles on TBR, focused post hoc comparisons were conducted for personal feedback (positive vs. negative, negative only, and positive only) and group feedback (positive vs. negative, negative only, and positive only). The results of these analyses are summarized below.

#### 3.3.1. Personal Feedback: Positive vs. Negative Feedback

When comparing the effects of positive and negative personal feedback within attachment styles, significant differences were found for the secure, anxiously attached, and avoidant groups. Securely attached individuals showed significantly lower TBR under negative feedback compared to positive feedback (*p* < 0.01, Cohen’s d = 0.76), indicating increased cognitive load in response to negative evaluation. Similarly, anxiously attached individuals exhibited significantly lower TBR under negative feedback (*p* < 0.01, Cohen’s d = 0.61). Avoidant individuals also exhibited significant differences (*p* < 0.05, Cohen’s d = 0.37), although the effect was less pronounced. These findings reflect a consistent sensitivity to feedback valence across these attachment styles, with negative personal feedback eliciting higher cognitive load. In contrast, fearful-avoidant individuals showed no significant differences (*p* > 0.05, Cohen’s d = 0.11), suggesting that their cognitive load remains relatively unaffected by the valence of personal feedback.

#### 3.3.2. Personal Feedback: Negative Feedback

When comparing the effects of negative personal feedback across attachment styles, significant differences were found between the secure group and all other attachment styles. Specifically, differences were observed between the secure and anxious groups (*p* < 0.01, Cohen’s d = 0.76), the secure and avoidant groups (*p* < 0.01, Cohen’s d = X.81), and the secure and fearful-avoidant groups (*p* < 0.01, Cohen’s d = 0.85). These findings indicate that secure individuals experience a distinct cognitive load compared to anxiously attached, avoidant, and fearful-avoidant individuals under negative feedback conditions. However, no significant differences were observed between anxiously attached, avoidant, and fearful-avoidant groups (*p* > 0.05), suggesting a relatively uniform cognitive load among insecure attachment profiles in response to negative feedback. Additionally, the effect sizes for these comparisons were small (Cohen’s d < 0.20), reinforcing the minimal differences between these groups.

#### 3.3.3. Personal Feedback: Positive Feedback

Under positive personal feedback, securely attached individuals exhibited distinct cognitive responses compared to all other attachment styles, including anxious (*p* < 0.01, Cohen’s d = 0.59), avoidant (*p* < 0.01, Cohen’s d = 0.62), and fearful-avoidant groups (*p* < 0.01, Cohen’s d = 0.73). Anxiously attached individuals also displayed significantly different responses compared to avoidant individuals (*p* < 0.05, Cohen’s d = 0.33) and fearful-avoidant individuals (*p* < 0.05, Cohen’s d = 0.42). However, avoidant and fearful-avoidant individuals showed no significant differences from each other (*p* > 0.05, Cohen’s d = 0.15), suggesting shared cognitive characteristics and reduced sensitivity to positive personal feedback.

#### 3.3.4. Group Feedback: Positive vs. Negative Feedback

Significant differences in TBR were found between positive and negative group feedback for securely attached individuals. Specifically, securely attached individuals showed significantly lower TBR under negative group feedback compared to positive feedback (*p* < 0.01, Cohen’s d = 0.52), reflecting an increased cognitive load in response to negative evaluation. In contrast, no significant differences were observed between positive and negative group feedback for anxiously attached, avoidant, and fearful-avoidant individuals (*p* > 0.05), and all corresponding effect sizes were small (Cohen’s d < 0.20). These findings suggest that, unlike securely attached individuals, insecurely attached individuals exhibited relatively stable cognitive load levels regardless of feedback valence in group feedback conditions.

#### 3.3.5. Group Feedback: Negative Feedback

When analyzing the effects of negative group feedback across attachment styles, significant differences emerged between securely attached individuals and each insecure attachment style. Specifically, securely attached individuals differed from anxiously attached individuals (*p* < 0.01, Cohen’s d = 0.57), avoidant individuals (*p* < 0.01, Cohen’s d = 0.59), and fearful-avoidant individuals (*p* < 0.01, Cohen’s d = 0.59), indicating that secure individuals processed negative group feedback differently. However, no significant differences were found among the anxiously attached, avoidant, and fearful-avoidant groups (*p* > 0.05), and all corresponding effect sizes were small (Cohen’s d < 0.20), suggesting that insecure individuals responded in a relatively similar manner to negative group feedback.

#### 3.3.6. Group Feedback: Positive Feedback

Under positive group feedback, securely attached individuals exhibited significantly different TBR responses compared to all insecure groups. Specifically, significant differences were observed between secure and anxiously attached individuals (*p* < 0.01, Cohen’s d = 0.38), secure and avoidant individuals (*p* < 0.01, Cohen’s d = 0.55), and secure and fearful-avoidant individuals (*p* < 0.01, Cohen’s d = 0.62). Similarly, anxiously attached individuals displayed significantly distinct TBR responses compared to avoidant individuals (*p* < 0.05, Cohen’s d = 0.34) and fearful-avoidant individuals (*p* < 0.05, Cohen’s d = 0.27). However, no significant differences were observed between avoidant and fearful-avoidant individuals (*p* > 0.05), and the corresponding effect size was small (Cohen’s d < 0.20), reinforcing the idea that these two attachment styles share cognitive characteristics, particularly in their reduced responsiveness to positive social evaluation.

#### 3.3.7. Summary of Post Hoc Comparisons

The post hoc comparisons reveal notable differences in how attachment styles respond to feedback. Secure and anxiously attached individuals demonstrate heightened sensitivity, with secure individuals consistently showing unique cognitive responses across feedback types and valences. Both secure and anxiously attached individuals exhibited distinct TBR responses under positive group feedback compared to avoidant and fearful-avoidant individuals, reinforcing their heightened engagement with social cues. A similar trend was observed under positive personal feedback, where secure and anxious individuals showed significantly different responses compared to avoidant groups. Negative feedback significantly increases cognitive load for both groups, reflecting their strong engagement with social cues. In contrast, avoidant and fearful-avoidant individuals exhibit reduced sensitivity, responding uniformly to feedback with minimal differentiation based on valence or context. These two groups share cognitive characteristics, particularly in their similar responses to negative and positive feedback, suggesting a tendency toward emotional disengagement. Additionally, post hoc comparisons revealed that secure and anxiously attached individuals showed more highly significant differences compared to avoidant individuals in personal feedback conditions, suggesting that their cognitive load responses are more reactive to feedback valence. Overall, secure and anxious styles are marked by active engagement, while avoidant and fearful-avoidant styles show detachment and reduced reactivity.

### 3.4. The Effect of Task Difficulty, Attachment Style, and Feedback on Cognitive Load

**Figure 6:**
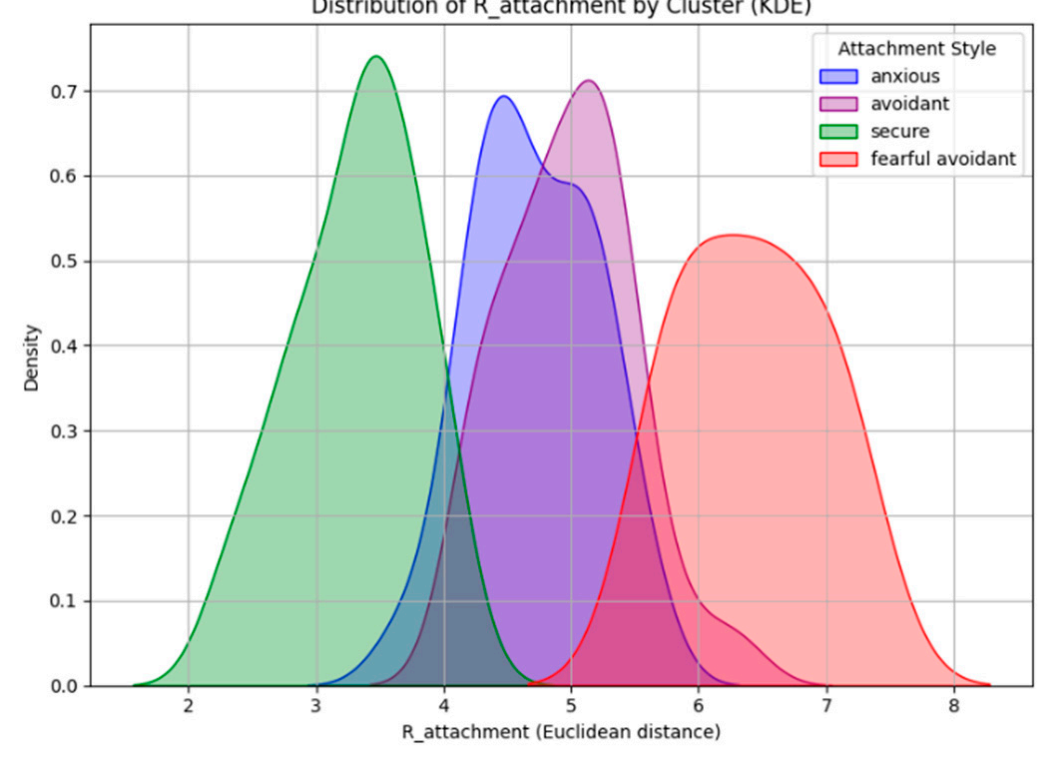
Distribution of R_attachment scores across attachment styles. Lower values indicate proximity to secure attachment, while higher values reflect greater deviation.

To better understand how task difficulty, attachment style, and feedback influence cognitive load, we introduced R_attachment as a continuous measure to complement the categorical attachment classifications. While attachment styles are often described as discrete categories, they exist on a spectrum defined by anxiety and avoidance (Laufer et al., 2024). R_attachment quantifies an individual’s position within this two-dimensional space, offering a more detailed representation of attachment tendencies. Figure 6 shows the distribution of R_attachment scores across attachment groups, illustrating how attachment styles vary along a continuous scale. Secure individuals have the lowest R_attachment values (M = 3.309, SD = 0.489), staying close to the origin, which represents balanced levels of anxiety and avoidance. Anxiously attached individuals (M = 4.707, SD = 0.479) and avoidant individuals (M = 4.979, SD = 0.501) have overlapping distributions, suggesting similarities in their attachment-related tendencies despite being distinct categories. Fearful-avoidant individuals have the highest R_attachment values (M = 6.416, SD = 0.555), reflecting the greatest distance from secure attachment. The figure shows a clear separation between secure and insecure attachment styles, while the overlap between anxious and avoidant groups suggests that attachment patterns exist on a spectrum rather than in rigid categories.

**Figure 7:**
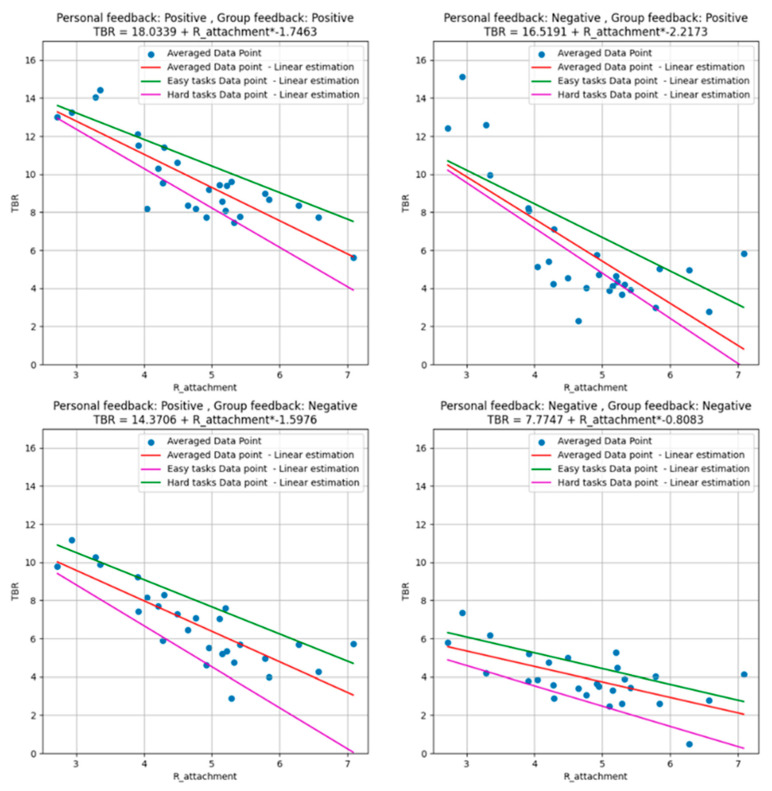
TBR as a function of R_attachment for different types of feedback, along with linear regressions (overall, hard, and easy games).

Figure 7 illustrates the relationship between TBR and R_attachment across four feedback conditions, highlighting how attachment insecurity modulates cognitive load under varying types of feedback and task difficulty. R_attachment, a composite measure derived from attachment-related anxiety and avoidance (Mizrahi et al., 2024a), consistently shows an inverse relationship with TBR in all panels, indicating greater cognitive load for individuals with higher attachment insecurity.

Task difficulty was determined by the difference in dot counts between the two sides. Easy tasks were defined as trials where the dot difference was 4 or 5, while difficult tasks were those with a difference of 1 or 2. Trials with a three-dot difference were included in the overall calculation but were not assigned to either the easy or difficult category. Each panel in Figure 7 includes three regression models: an overall model (red line), a model for difficult tasks (purple line), and a model for easy tasks (green line). We performed single-variable linear regression, where the regression slopes indicate the relationship between R_attachment and TBR across different task difficulties. All regression models, including the overall model and the models for specific task difficulties, were statistically significant at *p* < 0.01. These regression models consistently show decreasing TBR values with increasing R_attachment, with the slopes for difficult tasks reflecting greater cognitive strain across all feedback conditions.

In the personal-positive, group-positive condition (top left panel), TBR steadily decreases as R_attachment increases, with consistent patterns across overall, easy, and hard tasks. Even under uniformly positive feedback, individuals with higher attachment insecurity exhibit greater cognitive load, suggesting their heightened sensitivity to both personal and social evaluation. In the personal-negative, group-positive condition (top right panel), a steeper decline in TBR is observed, particularly for difficult tasks. Negative personal feedback appears to amplify cognitive load for individuals with higher R_attachment, even when accompanied by positive group feedback. This interaction indicates that conflicting feedback types impose additional strain, especially under challenging task conditions.

The personal-positive, group-negative condition (bottom left panel) follows a similar trend, with TBR decreasing as R_attachment increases. The effect of task difficulty is again pronounced, as hard tasks result in steeper declines in TBR compared to easier ones. This suggests that mixed feedback conditions, where affirming personal feedback contrasts with negative group feedback, exacerbate cognitive demands for individuals with insecure attachment styles. Finally, the personal-negative, group-negative condition (bottom right panel) demonstrates the steepest decline in TBR across all task types. The combined effect of dual negative feedback places the greatest cognitive load on individuals with higher attachment insecurity. The regression models for different task difficulty levels reveal the substantial cognitive strain induced by negative feedback in this context.

These findings highlight the interplay between attachment style, feedback type, and task difficulty. Across all conditions, individuals with higher R_attachment values consistently exhibit greater cognitive load, with harder tasks intensifying these effects. The significant results of both the overall and task-specific regression models (*p* < 0.01) reinforce the importance of attachment style in shaping cognitive and emotional responses to feedback scenarios.

**Figure 8:**
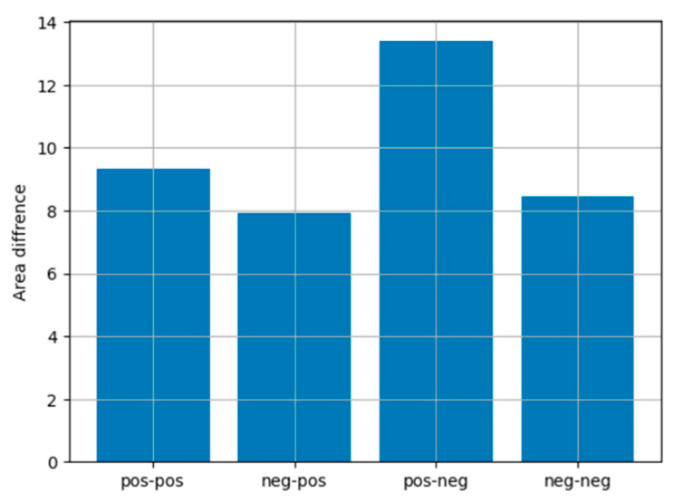
Area differences between regression models for hard and easy games across different feedback conditions. The feedback types are labeled on the *x*-axis (e.g., “pos-pos” indicates positive personal feedback and positive group feedback).

To explore the impact of task difficulty on cognitive load across varying feedback conditions, we calculated the area difference between the regression lines representing hard and easy tasks as a function of R_attachment. This metric reflects the cumulative divergence between the two models over the range of R_attachment values, offering a quantitative measure of how task difficulty modulates cognitive responses under different feedback scenarios. The assumption that changes in TBR correspond proportionally to variations in cognitive load is supported by previous findings, which demonstrate a systematic linear relationship between TBR and attentional control mechanisms across different task demands (Angelidis et al., 2018). Specifically, prior research has shown that TBR scales with cognitive effort in a predictable manner, reinforcing the validity of using regression-based area differences to assess the impact of task difficulty. The results of these calculations are presented in Figure 8.

The *x*-axis of Figure 8 represents the four feedback conditions: pos-pos (positive personal and positive group feedback), neg-pos (negative personal and positive group feedback), pos-neg (positive personal and negative group feedback), and neg-neg (negative personal and negative group feedback). The *y*-axis displays the calculated area differences, indicating the magnitude of the effect of task difficulty on TBR values. The largest area difference was observed for the pos-neg condition, suggesting that the discrepancy in cognitive load between hard and easy tasks is most pronounced when personal feedback is positive and group feedback is negative. This pattern highlights the significant cognitive demands imposed by mixed feedback, where the opposing valence of personal and group feedback appears to exacerbate task difficulty effects. Moderate area differences were found for the pos-pos and neg-neg conditions, where both personal and group feedback share the same valence. These results suggest that task difficulty impacts cognitive load less severely when feedback valence is consistent. The smallest area difference was observed in the neg-pos condition, indicating relatively stable cognitive load across difficulty levels.

## 4. Conclusions and Future Work

This study aimed to explore how attachment styles influence cognitive load under varying task difficulties and feedback conditions. By using the theta/beta ratio (TBR) as a neurophysiological marker, we examined how attachment-related emotional regulation and inhibitory control interact with feedback processing and task complexity. Two hypotheses were tested: (1) individuals with anxious attachment styles would exhibit heightened cognitive load in response to negative feedback due to their sensitivity to social punishment and tendency to ruminate on failures, and (2) avoidant individuals, relying on suppression strategies to manage stress, would show less responsiveness to positive feedback but experience increased cognitive strain as task complexity rose. In addition to these hypotheses, the study introduced R_attachment as a continuous measurement of attachment tendencies (Laufer et al., 2024), revealing that attachment insecurity exists along a spectrum rather than as rigid categories. Understanding how categorical attachment styles relate to R_attachment allowed us to integrate both traditional and dimensional perspectives into the cognitive load framework.

### 4.1. Feedback and Cognitive Load in Relation to Attachment

Negative personal feedback consistently led to elevated cognitive load across all attachment styles. Lower TBR values in this context indicate that self-critical evaluations require significant cognitive resources, particularly for insecurely attached individuals. Those with anxious attachment profiles demonstrated heightened reactivity to negative personal feedback, consistent with their sensitivity to social punishment and tendency to ruminate on perceived failures (Carnelley et al., 2007; Evraire et al., 2014; Gentzler et al., 2010; Hepper & Carnelley, 2012; Mikulincer & Orbach, 1995; Snyder et al., 2024; Vrtička et al., 2008).

The results confirm our hypotheses. Supporting hypothesis 1, anxiously attached individuals exhibited heightened cognitive load in response to negative personal feedback, reflecting their tendency to focus on social threat cues at the expense of task-relevant information (Basten et al., 2011; Chang, 2019; Van Emmichoven et al., 2003; Stanton & Campbell, 2015). Similarly, avoidant individuals demonstrated reduced responsiveness to positive feedback, consistent with hypothesis 2, but experienced increased cognitive strain as task complexity increased, suggesting that their suppression strategies are less effective under cognitive pressure (Edelstein & Gillath, 2008).

However, R_attachment analysis revealed additional complexity: although anxious and avoidant individuals are typically treated as distinct categories, Figure 6 demonstrated a substantial overlap between these groups (Gillath et al., 2005; Sirin, 2017) in terms of R_attachment distribution. This suggests that the cognitive load responses attributed to anxious or avoidant attachment may not be entirely separate, but instead reflect a shared dimension of attachment insecurity. The overlap between anxious and avoidant individuals in R_attachment distribution raises important considerations about attachment-based cognitive patterns. While categorical analyses in Figure 7 show distinct effects of attachment style on cognitive load, the continuous distribution in Figure 6 suggests that attachment-related cognitive responses may exist on a spectrum rather than within discrete categories (Laufer et al., 2024). This is particularly relevant for understanding why certain individuals classified as avoidant might still exhibit anxious-like cognitive load patterns under specific conditions (Mikulincer et al., 2004). Thus, while categorical attachment classifications remain useful, integrating dimensional perspectives through R_attachment provides a more comprehensive understanding of how feedback modulates cognitive engagement across different attachment tendencies.

When examining the role of group feedback, additional patterns emerged. Avoidant individuals demonstrated lower cognitive load in response to positive group feedback, likely reflecting their strategy of disengaging from social comparisons and focusing on internal evaluations as a way to reduce stress. Fearful-avoidant individuals exhibited cognitive responses to positive feedback that closely resembled those of avoidant individuals, characterized by reduced sensitivity to positive reinforcement and a tendency to disengage from social evaluation (Hepper & Carnelley, 2012). However, unlike avoidant individuals, fearful-avoidant individuals exhibited greater cognitive strain in response to negative group feedback, likely due to their heightened sensitivity to both social comparison and self-critical tendencies (Dan et al., 2020). These findings suggest that avoidant and fearful-avoidant individuals share a detached response pattern to positive social feedback, while only fearful-avoidant individuals experience increased stress under negative group evaluation. Feedback strategies for fearful-avoidant individuals should focus on reducing both comparative stress and the emotional weight of personal evaluations to address their heightened sensitivity (Hepper & Carnelley, 2012; Philipp-Muller & MacDonald, 2017; Schwartz et al., 2007; Smith LeBeau & Buckingham, 2008).

### 4.2. Task Difficulty and Cognitive Load

Task difficulty significantly influenced cognitive load, with insecure attachment styles exacerbating the effect of increased complexity. Securely attached individuals demonstrated resilience, maintaining relatively stable TBR values across task difficulties. This stability reflects stronger inhibitory control mechanisms that enable effective allocation of cognitive resources even under stress. Conversely, individuals with insecure attachment styles, particularly those with anxious or fearful-avoidant profiles, exhibited marked increases in cognitive load as task complexity rose. These findings validate the hypothesis that insecure attachment impairs the regulation of cognitive demands, as inhibitory control mechanisms become overwhelmed in stressful environments (Lim et al., 2020; Silva et al., 2012; Simpson & Rholes, 2017; Warren et al., 2010).

Further supporting hypothesis 2, the study found that increases in cognitive load due to task difficulty followed a linear trend with respect to R_attachment. Individuals higher in attachment insecurity (i.e., with higher R_attachment values) exhibited the most pronounced cognitive strain when transitioning from easy to difficult tasks. The findings suggest that while avoidant individuals attempt to disengage from feedback and regulate cognitive resources through suppression, their ability to do so diminishes as task demands increase. Fearful-avoidant individuals, in particular, experienced the highest cognitive load under difficult conditions, reflecting their compounded vulnerability, combining heightened sensitivity to stress with impaired regulatory strategies. These results reinforce the idea that attachment insecurity amplifies cognitive strain in high-demand situations, aligning with prior research on stress regulation and attentional control deficits in insecure attachment (Gillath et al., 2005; Kashdan et al., 2008).

### 4.3. Implications for Emotional Regulation and Feedback

The study’s results demonstrate how attachment styles influence the processing of feedback and the management of cognitive demands. Securely attached individuals showed the capacity to engage constructively with feedback, distributing cognitive resources efficiently while maintaining emotional balance. In contrast, individuals with insecure attachment styles struggled to manage emotional responses, leading to heightened cognitive load and diminished task performance. For example, anxiously attached individuals exhibited heightened sensitivity to negative feedback, while avoidantly attached individuals, who often rely on emotional suppression, found this strategy less effective as task complexity increased (Gillath et al., 2005).

Negative personal feedback emerged as a particularly significant driver of cognitive load, often outweighing the effects of group feedback. Among insecurely attached individuals, the emotional burden associated with perceived personal failure consumed substantial cognitive resources, overshadowing external evaluations. This suggests that negative personal feedback can trigger self-critical thoughts, making it harder to process external feedback constructively. These findings emphasize the importance of tailoring feedback strategies to suit individual attachment profiles, as discussed in the introduction. The analysis also highlights how feedback valence interacts with task difficulty to shape cognitive load. Feedback with consistent valence generally mitigated the cognitive strain of more demanding tasks. Notably, the smallest area difference was observed in the neg-pos condition, where negative personal feedback was paired with positive group feedback. This combination appeared to stabilize cognitive load, likely because positive group feedback offset the emotional strain caused by negative personal feedback.

In contrast, the pos-neg condition, which combined positive personal feedback with negative group feedback, did not exhibit the same stabilizing effect. Negative group feedback, particularly in public contexts, may have heightened stress and defensiveness, reducing the positive impact of personal feedback (Crost et al., 2008; Czekalla et al., 2021; Y. Zhang et al., 2023). This finding highlights the disproportionate impact of negative group feedback on cognitive load compared to its positive counterpart.

Consistent feedback valence, as observed in the pos-pos and neg-neg conditions, resulted in moderate area differences. These results suggest that uniform feedback can help set clear expectations, reducing variability in cognitive load. Overall, these insights underline the importance of designing feedback strategies that carefully balance consistency and variability to optimize cognitive engagement, particularly in contexts requiring sustained focus and adaptability.

### 4.4. Implications for Adaptive Systems and Feedback Design

The results of this study provide valuable insights for designing adaptive systems and improving human–agent interactions. Personal feedback, particularly negative feedback, consistently increased cognitive load across all attachment profiles compared to positive personal feedback. However, group feedback elicited more varied responses depending on attachment style. Avoidant and fearful-avoidant individuals showed a reversal effect, where negative group feedback heightened cognitive load. For avoidant individuals, this pattern likely reflects their preference to disengage from group-based evaluations, as such feedback challenges their reliance on emotional suppression. Fearful-avoidant individuals exhibited an even stronger reaction, with negative group feedback amplifying cognitive strain due to their heightened sensitivity to both personal and social stressors (Chen & Deng, 2024; Vrtička et al., 2008).

These findings emphasize the importance of tailoring feedback strategies to individual differences in emotional regulation. Avoidant and fearful-avoidant individuals may benefit from personalized feedback that prioritizes individual progress and positive reinforcement, reducing the cognitive strain associated with social evaluations. By contrast, securely attached individuals demonstrated greater flexibility, maintaining stable cognitive load levels across feedback types and task complexities. This resilience enables them to benefit from a broader range of feedback strategies, including group-based approaches that encourage collaboration or competition (Bernecker et al., 2014; Markin & Marmarosh, 2010).

The findings related to R_attachment offer additional insights for adaptive systems. By treating attachment tendencies as a spectrum rather than rigid categories, future feedback mechanisms could be tailored to individuals based on their specific attachment-related cognitive regulation strategies. Individuals with high R_attachment values, for instance, might benefit from gradual exposure to evaluative feedback, allowing them to process it incrementally rather than experiencing abrupt shifts in cognitive load. Additionally, real-time cognitive monitoring using EEG-based indicators such as TBR fluctuations could detect moments when individuals are at risk of cognitive overload, enabling adaptive feedback strategies that mitigate stress and optimize task performance. These insights could be directly integrated into EEG-based neurofeedback systems, which dynamically adjust task complexity or feedback strategies in response to cognitive strain. Such personalized interventions hold valuable applications in settings such as education, workplace training, and other environments that demand prolonged cognitive effort (El Kerdawy et al., 2020; Yoo et al., 2023).

### 4.5. Limitations and Future Directions

This study sheds light on how attachment styles, feedback, and cognitive load interact, but several limitations warrant consideration. First, although proportional allocation was used to improve the representativeness of the sample, the predominantly student participant pool presented limitations in diversity. Key demographic factors such as age, socioeconomic status, and cultural background were underrepresented, which may influence attachment styles (Brown et al., 2008; David et al., 2020; Taylor et al., 2015). This limited diversity could affect the broader applicability of the findings. Future research should aim to include participants from more varied demographic groups and adopt longitudinal designs to explore how attachment styles develop and evolve across different populations.

Second, while the use of EEG-based measures of cognitive load was insightful, it may have missed other valuable physiological and behavioral indicators (Ahmadi et al., 2024; Gokay et al., 2015; Macatee et al., 2017; Mather & Thayer, 2018; Zhu et al., 2019). Incorporating additional methods, such as heart rate variability (HRV), skin conductance, or eye tracking, could provide a more comprehensive understanding of the connections between stress, emotional regulation, and cognitive load.

Third, this study adopted a consistent mapping of feedback modalities, using smiley icons exclusively for group feedback and written text for personal feedback. This design choice was informed by findings suggesting that graphical feedback can enhance engagement and foster collaboration in group contexts (Boyle et al., 2017). Written feedback, meanwhile, demonstrated utility in delivering tailored, actionable information in individualized interventions, such as smoking cessation programs (Becoña & Vázquez, 2001). While this approach aimed to optimize clarity and engagement, the lack of counterbalancing between feedback modalities and contexts may have introduced confounding effects. Specifically, it remains unclear whether the observed results were driven by the content of the feedback (group vs. personal) or its modality (icon-based vs. written).

Prior research underscores the importance of tailoring feedback to task goals and user characteristics, suggesting that future studies systematically vary both content and modality to disentangle these effects (Hysong, 2009). Additionally, feedback presented in public versus private contexts may elicit differential responses due to identity concerns and group image management. Public group feedback can heighten defensiveness, especially when critical, while private feedback tends to encourage reflection and constructive action (Rabinovich & Morton, 2017). While this aligns with the broader concept of group feedback, our study specifically defines group feedback as performance-based information reflecting the group’s performance rather than feedback given in a public setting. This distinction clarifies that group feedback can influence cognitive and emotional responses through mechanisms beyond public scrutiny. Future research should explore these dynamics to refine feedback strategies and enhance their applicability across diverse settings.

Fourth, this study identified significant two-way and three-way interactions involving attachment class, group feedback, and personal feedback. However, the primary focus was on two-way interactions: attachment class × group feedback and attachment class × personal feedback, aligning with the study’s central hypotheses. By not fully exploring the three-way interaction, potential conditional effects may have been overlooked. For example, avoidant individuals might respond differently to group feedback when paired with specific types of personal feedback, and anxiously attached individuals may exhibit heightened sensitivity to personal feedback, irrespective of group feedback. Future studies should consider systematically examining these more complex patterns to gain deeper insights into the interplay between feedback types and attachment styles.

Finally, another potential limitation of our study is the differing criteria for individual and group feedback, with individual feedback based on accuracy and group feedback based on response speed. This introduces a possible confound, as the speed–accuracy tradeoff (SAT) suggests that participants may adjust their response strategy depending on whether speed or accuracy is emphasized (Heitz, 2014). Specifically, participants may prioritize faster responses in the group feedback condition while focusing on accuracy in the individual feedback condition, potentially influencing cognitive load and engagement. However, our design mitigated extreme tradeoff effects by enforcing a strict 1 s response window, preventing excessive emphasis on either speed or accuracy. Despite this control, future studies should consider feedback designs that equate criteria across conditions to fully isolate the effects of individual versus group feedback.

Future studies should also investigate how attachment styles influence cognitive load in real-world settings (e.g., (Hsin-Ke et al., 2018; Riley, 2009)). Expanding research to include dynamic tasks such as team-based problem solving or social decision making in professional or educational environments could provide deeper insights into how attachment-related differences affect performance and emotional responses in everyday situations. Such work could help uncover strategies for improving collaboration, reducing stress, and enhancing performance in group settings.

Moreover, future studies could explore mixed-effects linear regression models to further investigate interaction effects between attachment style, feedback type, and task difficulty on TBR. While our current approach used factorial ANOVA to systematically assess interactions, mixed-effects models could provide an alternative framework for examining within-participant variations across conditions. Additionally, more advanced machine learning methods, such as tree-based models, may allow for hierarchical representations of attachment-related cognitive load patterns.

Emerging technologies, including machine learning, open up exciting opportunities for future exploration. These tools could help researchers analyze the complex interactions between attachment styles, task difficulty, and feedback types, offering more precise predictions of cognitive and emotional responses. Adaptive systems informed by these findings could adjust feedback or task designs in real time to better suit individual needs. For example, integrating real-time data from EEG or other physiological measures could support personalized interventions to lower cognitive strain, improve emotional regulation, and boost engagement across various domains, such as education and workplace training (D’Urso et al., 2024; De Filippi et al., 2021; Pejanović & Radaković, 2024; Zotev et al., 2014). Building on these findings will require combining a variety of methods, expanding the scope to practical applications, and harnessing computational tools to tailor strategies to individual differences. By doing so, future research can contribute to developing systems and interventions that enhance performance and well-being in diverse settings.

## Data Availability

All the experimental data, which includes the players’ electrophysiological recordings and the corresponding coordination logs, are stored on the servers of Ariel University. The data can be obtained by request from The IRB member, Chen Hajaj (chenha@ariel.ac.il) or from one of the authors (Dor Mizrahi dor.mizrahi1@msmail.ariel.ac.il, Ilan Laufer ilanl@ariel.ac.il, Inon Zuckerman inonzu@ariel.ac.il).

## References

[B1-behavsci-15-00427] Ahmad M. I., Robb D. A., Keller I., Lohan K. (2020). Towards a multimodal measure for physiological behaviours to estimate cognitive load. Engineering psychology and cognitive ergonomics. Mental workload, human physiology, and human energy.

[B2-behavsci-15-00427] Ahmadi N. K., Ozgur S. F., Kiziltan E. (2024). Evaluating the effects of different cognitive tasks on autonomic nervous system responses: Implementation of a high–precision, low–cost complementary method. Brain and Behavior.

[B3-behavsci-15-00427] Angelidis A., Hagenaars M., van Son D., van der Does W., Putman P. (2018). Do not look away! Spontaneous frontal EEG theta/beta ratio as a marker for cognitive control over attention to mild and high threat. Biological Psychology.

[B4-behavsci-15-00427] Angelidis A., van der Does W., Schakel L., Putman P. (2016). Frontal EEG theta/beta ratio as an electrophysiological marker for attentional control and its test-retest reliability. Biological Psychology.

[B5-behavsci-15-00427] Archer-Kath J., Johnson D. W., Johnson R. T. (1994). Individual versus group feedback in cooperative groups. Journal of Social Psychology.

[B6-behavsci-15-00427] Bar-Haim Y., Lamy D., Pergamin L., Bakermans-Kranenburg M. J., van IJzendoorn M. H. (2007). Threat-related attentional bias in anxious and nonanxious individuals: A meta-analytic study. Psychological Bulletin.

[B7-behavsci-15-00427] Basten U., Stelzel C., Fiebach C. J. (2011). Trait anxiety modulates the neural efficiency of inhibitory control. Journal of Cognitive Neuroscience.

[B8-behavsci-15-00427] Becoña E., Vázquez F. L. (2001). Effectiveness of personalized written feedback through a mail intervention for smoking cessation: A randomized-controlled trial in Spanish smokers. Journal of Consulting and Clinical Psychology.

[B9-behavsci-15-00427] Bendezú J. J., Loughlin-Presnal J. E., Wadsworth M. E. (2019). Attachment security moderates effects of uncontrollable stress on preadolescent hypothalamic–pituitary–adrenal axis responses: Evidence of regulatory fit. Psychological Science.

[B10-behavsci-15-00427] Bernecker S. L., Levy K. N., Ellison W. D. (2014). A meta-analysis of the relation between patient adult attachment style and the working alliance. Psychotherapy Research.

[B11-behavsci-15-00427] Bowlby J. (1969). Attachment and loss v. 3.

[B12-behavsci-15-00427] Boyle S. C., Earle A. M., LaBrie J. W., Smith D. J. (2017). PNF 2.0? Initial evidence that gamification can increase the efficacy of brief, web-based personalized normative feedback alcohol interventions. Addictive Behaviors.

[B13-behavsci-15-00427] Brown D., Rodgers Y. H., Kapadia K. (2008). Multicultural considerations for the application of attachment theory. American Journal of Psychotherapy.

[B14-behavsci-15-00427] Buchheim A., George C., Gündel H., Viviani R. (2017). Neuroscience of human attachment. Frontiers in Human Neuroscience.

[B15-behavsci-15-00427] Carnelley K. B., Israel S., Brennan K. A. (2007). The role of attachment in influencing reactions to manipulated feedback from romantic partners. European Journal of Social Psychology.

[B16-behavsci-15-00427] Chang C. (2019). Ambivalent Facebook users: Anxious attachment style and goal cognition. Journal of Social and Personal Relationships.

[B17-behavsci-15-00427] Chen Y., Deng X. (2024). How socially avoidant emerging adults process social feedback during human-to-human interaction after social rejection: An event-related potential study. Behavioral Sciences.

[B18-behavsci-15-00427] Comte A., Szymanskaa M., Monnin J., Moulina T., Nezelofa S., Magnin E., Jardrie R., Vulliez-Coadya L. (2024). Neural correlates of distress and comfort in individuals with avoidant, anxious and secure attachment style: An fMRI study. Attachment & Human Development.

[B19-behavsci-15-00427] Crost N. W., Pauls C. A., Wacker J. (2008). Defensiveness and anxiety predict frontal EEG asymmetry only in specific situational contexts. Biological Psychology.

[B20-behavsci-15-00427] Cushing T., Robertson S., Mannes J., Marshall N., Carey M. J., Duschinsky R., Meiser-Stedman R. (2024). The relationship between attachment and posttraumatic stress in children and adolescents: A meta-analytic review. Development and Psychopathology.

[B21-behavsci-15-00427] Czekalla N., Stierand J., Stolz D. S., Mayer A. V., Voges J. F., Rademacher L., Paulus F. M., Krach S., Müller-Pinzler L. (2021). Self-beneficial belief updating as a coping mechanism for stress-induced negative affect. Scientific Reports.

[B22-behavsci-15-00427] Dan O., Zreik G., Raz S. (2020). The relationship between individuals with fearful-avoidant adult attachment orientation and early neural responses to emotional content: An event-related potentials (ERPs) study. Neuropsychology.

[B23-behavsci-15-00427] David M. E., Carter K., Alvarez C. (2020). An assessment of attachment style measures in marketing. European Journal of Marketing.

[B33-behavsci-15-00427] De Filippi E., Wolter M., Pastor de Melo B. R., Tierra-Criollo C. J., Bortolini T., Deco G., Moll J. (2021). Classification of complex emotions using EEG and virtual environment: Proof of concept and therapeutic implication. Frontiers in Human Neuroscience.

[B24-behavsci-15-00427] Delorme A., Makeig S. (2004). EEGLAB: An open source toolbox for analysis of single-trial EEG dynamics including independent component analysis. Journal of Neuroscience Methods.

[B25-behavsci-15-00427] D’Urso S., Luongo R., Sciarrone F. (2024). Enhancing educational outcomes through eeg-based cognitive indices and supervised machine learning: A methodological framework. 2024 28th International Conference Information Visualisation (IV).

[B26-behavsci-15-00427] Dykas M. J., Cassidy J. (2011). Attachment and the processing of social information across the life span: Theory and evidence. Psychological Bulletin.

[B27-behavsci-15-00427] Edelstein R. S. (2006). Attachment and emotional memory: Investigating the source and extent of avoidant memory impairments. Emotion.

[B28-behavsci-15-00427] Edelstein R. S., Gillath O. (2008). Avoiding interference: Adult attachment and emotional processing biases. Personality and Social Psychology Bulletin.

[B29-behavsci-15-00427] Ein-Dor T., Mikulincer M., Shaver P. R. (2011). Attachment insecurities and the processing of threat-related information: Studying the schemas involved in insecure people’s coping strategies. Journal of Personality and Social Psychology.

[B30-behavsci-15-00427] El Kerdawy M., El Halaby M., Hassan A., Maher M., Fayed H., Shawky D., Badawi A. (2020). The automatic detection of cognition using eeg and facial expressions. Sensors.

[B32-behavsci-15-00427] Evraire L. E., Ludmer J. A., Dozois D. J. (2014). The influence of priming attachment styles on excessive reassurance seeking and negative feedback seeking in depression. Journal of Social and Clinical Psychology.

[B34-behavsci-15-00427] Gentzler A. L., Kerns K. A., Keener E. (2010). Emotional reactions and regulatory responses to negative and positive events: Associations with attachment and gender. Motivation and Emotion.

[B35-behavsci-15-00427] Gillath O., Bunge S. A., Shaver P. R., Wendelken C., Mikulincer M. (2005). Attachment-style differences in the ability to suppress negative thoughts: Exploring the neural correlates. Neuroimage.

[B36-behavsci-15-00427] Gillath O., Giesbrecht B., Shaver P. R. (2009). Attachment, attention, and cognitive control: Attachment style and performance on general attention tasks. Journal of Experimental Social Psychology.

[B37-behavsci-15-00427] Gokay R., Masazade E., Aydin C., Barkana D. E. (2015). Emotional state and cognitive load analysis using features from BVP and SC sensors. 2015 IEEE International Conference on Multisensor Fusion and Integration for Intelligent Systems (MFI).

[B38-behavsci-15-00427] Heitz R. P. (2014). The speed-accuracy tradeoff: History, physiology, methodology, and behavior. Frontiers in Neuroscience.

[B39-behavsci-15-00427] Hepper E. G., Carnelley K. B. (2012). The self–esteem roller coaster: Adult attachment moderates the impact of daily feedback. Personal Relationships.

[B40-behavsci-15-00427] Hsin-Ke L., Peng-Chun L., Cheng-Chih L. (2018). A study of the effect of learning attachment type on collaborative learning activity-a case of network course. 2nd International Conference on E-Society, E-Education and E-Technology.

[B41-behavsci-15-00427] Hysong S. J. (2009). Meta-analysis: Audit and feedback features impact effectiveness on care quality. Medical Care.

[B42-behavsci-15-00427] Hyvärinen A., Oja E. (2000). Independent component analysis: Algorithms and applications. Neural Networks.

[B43-behavsci-15-00427] Jain A. K. (2008). Data clustering: 50 years beyond K-means. Machine learning and knowledge discovery in databases.

[B44-behavsci-15-00427] Karreman A., Vingerhoets A. J. (2019). Attachment styles and secure base priming in relation to emotional reactivity after frustration induction. Cognition and Emotion.

[B45-behavsci-15-00427] Kashdan T. B., Zvolensky M. J., McLeish A. C. (2008). Anxiety sensitivity and affect regulatory strategies: Individual and interactive risk factors for anxiety-related symptoms. Journal of Anxiety Disorders.

[B46-behavsci-15-00427] Khazi M., Kumar A., MJ V. (2012). Analysis of EEG using 10: 20 electrode system. International Journal of Innovative Research in Science, Engineering and Technology.

[B47-behavsci-15-00427] Kim J. J., Kent K. M., Cunnington R., Gilbert P., Kirby J. N. (2020). Attachment styles modulate neural markers of threat and imagery when engaging in self-criticism. Scientific Reports.

[B48-behavsci-15-00427] Lange C., Costley J., Han S. (2017). The effects of extraneous load on the relationship between self-regulated effort and germane load within an e-learning environment. International Review of Research in Open and Distributed Learning.

[B49-behavsci-15-00427] Laufer I., Mizrahi D., Zuckerman I. (2022). An electrophysiological model for assessing cognitive load in tacit coordination games. Sensors.

[B50-behavsci-15-00427] Laufer I., Mizrahi D., Zuckerman I. (2024). Enhancing eeg-based attachment style prediction: Unveiling the impact of feature domains. Frontiers in Psychology.

[B51-behavsci-15-00427] Leyh R., Heinisch C., Kungl M. T., Spangler G. (2016). Attachment representation moderates the influence of emotional context on information processing. Frontiers in Human Neuroscience.

[B52-behavsci-15-00427] Lim B. H., Hodges M. A., Lilly M. M. (2020). The differential effects of insecure attachment on post-traumatic stress: A systematic review of extant findings and explanatory mechanisms. Trauma, Violence, & Abuse.

[B53-behavsci-15-00427] Liu Y., Ding Y., Lu L., Chen X. (2017). Attention bias of avoidant individuals to attachment emotion pictures. Scientific Reports.

[B54-behavsci-15-00427] Liu Y., Yu Y., Ye Z., Li M., Zhang Y., Zhou Z. (2023). Fusion of spatial, temporal, and spectral EEG signatures improves multilevel cognitive load prediction. IEEE Transactions on Human-Machine Systems.

[B55-behavsci-15-00427] Lu H. J., Lansford J. E., Liu Y. Y., Chen B. B., Bornstein M. H., Skinner A. T., Dodge K. A., Steinberg L., Deater-Deckard K., Rothenberg W. A., Bacchini D., Pastorelli C., Alampay L. P., Sorbring E., Gurdal S., Al-Hassan S. M., Oburu P., Yotanyamaneewong S., Tapanya S., Chang L. (2024). Attachment security, environmental adversity, and fast life history behavioral profiles in human adolescents. Development and Psychopathology.

[B56-behavsci-15-00427] Macatee R. J., Albanese B. J., Schmidt N. B., Cougle J. R. (2017). The moderating influence of heart rate variability on stressor-elicited change in pupillary and attentional indices of emotional processing: An eye-Tracking study. Biological Psychology.

[B57-behavsci-15-00427] Magai C., Cohen C., Milburn N., Thorpe B., McPherson R., Peralta D. (2001). Attachment styles in older European American and African American adults. The Journals of Gerontology Series B: Psychological Sciences and Social Sciences.

[B58-behavsci-15-00427] Markin R. D., Marmarosh C. (2010). Application of adult attachment theory to group member transference and the group therapy process. Psychotherapy: Theory, Research, Practice, Training.

[B59-behavsci-15-00427] Mather M., Thayer J. F. (2018). How heart rate variability affects emotion regulation brain networks. Current Opinion in Behavioral Sciences.

[B60-behavsci-15-00427] Mikulincer M. (1997). Adult attachment style and information processing: Individual differences in curiosity and cognitive closure. Journal of Personality and Social Psychology.

[B61-behavsci-15-00427] Mikulincer M., Dolev T., Shaver P. R. (2004). Attachment-related strategies during thought suppression: Ironic rebounds and vulnerable self-representations. Journal of Personality and Social Psychology.

[B62-behavsci-15-00427] Mikulincer M., Orbach I. (1995). Attachment styles and repressive defensiveness: The accessibility and architecture of affective memories. Journal of Personality and Social Psychology.

[B63-behavsci-15-00427] Mizrahi D., Laufer I., Zuckerman I. (2024a). Comparative analysis of ROCKET-driven and classic EEG features in predicting attachment styles. BMC Psychology.

[B64-behavsci-15-00427] Mizrahi D., Laufer I., Zuckerman I. (2024b). Neurophysiological insights into sequential decision-making: Exploring the secretary problem through ERPs and TBR dynamics. BMC Psychology.

[B65-behavsci-15-00427] Nezhad F. G., Mirmohammad M. S., Rostami R., Ahmadi H. (2025). Comparison of theta beta ratio in children with attention deficit/hyperactive disorder and specific learning disorder during active EEG. Basic and Clinical Neuroscience.

[B66-behavsci-15-00427] Ogle C. M., Cozza S. J., Kelle H., Bard K. A. (2017). Contemporary applications of attachment theory: A Review of the cultural nature of attachment: Contextualizing relationships and development. The cultural nature of attachment contextualizing relationships and development.

[B67-behavsci-15-00427] Orru G., Longo L. (2019). The evolution of cognitive load theory and the measurement of its intrinsic, extraneous and germane loads: A review. Human Mental Workload: Models and Applications: Second International Symposium, H-WORKLOAD 2018.

[B68-behavsci-15-00427] Pejanović V., Radaković M. (2024). Classification of cognitive stress as a psychological indicator through machine learning. Science International Journal.

[B69-behavsci-15-00427] Peng X., Gillath O., Jiang M., Wang B., Zhang J., Wu L. (2024). Attachment style and attention bias to emotional information: The moderating effect of stress, stimulus characteristics, and attention stage. Journal of Personality.

[B70-behavsci-15-00427] Pester B., Ligges C. (2018). Does independent component analysis influence EEG connectivity analyses?. Engineering in Medicine and Biology Society (EMBC).

[B71-behavsci-15-00427] Philipp-Muller A., MacDonald G. (2017). Avoidant individuals may have muted responses to social warmth after all: An attempted replication of MacDonald and Borsook (2010). Journal of Experimental Social Psychology.

[B72-behavsci-15-00427] Putman P., Verkuil B., Arias-Garcia E., Pantazi I., van Schie C. (2014). EEG theta/beta ratio as a potential biomarker for attentional control and resilience against deleterious effects of stress on attention. Cognitive, Affective, & Behavioral Neuroscience.

[B73-behavsci-15-00427] Rabinovich A., Morton T. A. (2017). Things we (don’t) want to hear: Exploring responses to group-based feedback. European Review of Social Psychology.

[B74-behavsci-15-00427] Ran G., Zhang Q. (2018). The neural correlates of attachment style during emotional processing: An activation likelihood estimation meta-analysis. Attachment & Human Development.

[B75-behavsci-15-00427] Renard Y., Lotte F., Gibert G., Congedo M., Maby E., Delannoy V., Bertrand O., Le’cuyer A. (2010). Openvibe: An open-source software platform to design, test, and use brain–computer interfaces in real and virtual environments. Presence: Teleoperators and Virtual Environments.

[B76-behavsci-15-00427] Riley P. (2009). An adult attachment perspective on the student–teacher relationship & classroom management difficulties. Teaching and Teacher Education.

[B77-behavsci-15-00427] Schwartz J. P., Lindley L. D., Buboltz W. C. (2007). Adult attachment orientations: Relation to affiliation motivation. Counselling Psychology Quarterly.

[B78-behavsci-15-00427] Seufert T., Jänen I., Brünken R. (2007). The impact of intrinsic cognitive load on the effectiveness of graphical help for coherence formation. Computers in Human Behavior.

[B79-behavsci-15-00427] Sibley C. G., Fischer R., Liu J. H. (2005). Reliability and validity of the revised experiences in close relationships (ECR-R) self-report measure of adult romantic attachment. Personality and Social Psychology Bulletin.

[B80-behavsci-15-00427] Sibley C. G., Liu J. H. (2004). Short-term temporal stability and factor structure of the revised experiences in close relationships (ECR-R) measure of adult attachment. Personality and Individual Differences.

[B81-behavsci-15-00427] Silva C., Soares I., Esteves F. (2012). Attachment insecurity and strategies for regulation: When emotion triggers attention. Scandinavian Journal of Psychology.

[B82-behavsci-15-00427] Simpson J. A., Rholes W. S. (2017). Adult attachment, stress, and romantic relationships. Current Opinion in Psychology.

[B83-behavsci-15-00427] Sirin H. D. (2017). The predictive power of adult attachment patterns on interpersonal cognitive distortions of university students. Educational Research and Reviews.

[B84-behavsci-15-00427] Smith LeBeau L., Buckingham J. T. (2008). Relationship social comparison tendencies, insecurity, and perceived relationship quality. Journal of Social and Personal Relationships.

[B85-behavsci-15-00427] Snyder M. D., Sun E. R., Nie J., Jakubiak B. K. (2024). When and how do people share? Attachment anxiety predicts support–seeking strategies. Personal Relationships.

[B86-behavsci-15-00427] Stanton S. C., Campbell L. (2015). Can’t get you off my mind: Relationship reflection creates cognitive load for more anxiously attached individuals. Journal of Social and Personal Relationships.

[B87-behavsci-15-00427] Stiller K. D., Schworm S. (2019). Game-based learning of the structure and functioning of body cells in a foreign language: Effects on motivation, cognitive load, and performance. Frontiers in Education.

[B88-behavsci-15-00427] Stout D. M., Shackman A. J., Pedersen W. S., Miskovich T. A., Larson C. L. (2017). Neural circuitry governing anxious individuals’ mis-allocation of working memory to threat. Scientific Reports.

[B89-behavsci-15-00427] Suzuki Y., Wild F., Scanlon E. (2024). Measuring cognitive load in augmented reality with physiological methods: A systematic review. Journal of Computer Assisted Learning.

[B90-behavsci-15-00427] Taylor P., Rietzschel J., Danquah A., Berry K. (2015). Changes in attachment representations during psychological therapy. Psychotherapy Research.

[B91-behavsci-15-00427] Tindale R. S., Kulik C. T., Scott L. A. (1991). Individual and group feedback and performance: An attributional perspective. Basic and Applied Social Psychology.

[B92-behavsci-15-00427] van der Horst F. C., van Rosmalen L., van der Veer R. (2024). The American contribution to attachment theory: John Bowlby’s WHO trip to the USA in 1950 and the development of his ideas on separation and attachment. Attachment & Human Development.

[B31-behavsci-15-00427] Van Emmichoven I. A. Z., Van Ijzendoorn M. H., De Ruiter C., Brosschot J. F. (2003). Selective processing of threatening information: Effects of attachment representation and anxiety disorder on attention and memory. Development and Psychopathology.

[B93-behavsci-15-00427] Vigário R., Sarela J., Jousmiki V., Hämäläinen M., Oja E. (2000). Independent component approach to the analysis of EEG and MEG recordings. IEEE Transactions on Biomedical Engineering.

[B94-behavsci-15-00427] Vrtička P., Andersson F., Grandjean D., Sander D., Vuilleumier P. (2008). Individual attachment style modulates human amygdala and striatum activation during social appraisal. PLoS ONE.

[B95-behavsci-15-00427] Warren S. L., Bost K. K., Roisman G. I., Silton R., Spielberg J. M., Engels A. S., Choi E., Sutton B. P., Miller G. A., Heller W. (2010). Effects of adult attachment and emotional distractors on brain mechanisms of cognitive control. Psychological Science.

[B96-behavsci-15-00427] Yoo G., Kim H., Hong S. (2023). Prediction of cognitive load from electroencephalography signals using long short-term memory network. Bioengineering.

[B97-behavsci-15-00427] Zhang D.-W., Li H., Wu Z., Zhao Q., Song Y., Liu L., Qian Q., Wang Y., Roodenrys S., Johnstone S. J., De Blasio F. M., Sun L. (2019). Electroencephalogram theta/beta ratio and spectral power correlates of executive functions in children and adolescents with AD/HD. Journal of Attention Disorders.

[B98-behavsci-15-00427] Zhang Y., Li Y., Mai X. (2023). Fear of negative evaluation modulates the processing of social evaluative feedback with different valence and contexts. Cerebral Cortex.

[B99-behavsci-15-00427] Zhu J., Ji L., Liu C. (2019). Heart rate variability monitoring for emotion and disorders of emotion. Physiological Measurement.

[B100-behavsci-15-00427] Zotev V., Phillips R., Yuan H., Misaki M., Bodurka J. (2014). Self-regulation of human brain activity using simultaneous real-time fMRI and EEG neurofeedback. NeuroImage.

[B101-behavsci-15-00427] Zuckerman I., Mizrahi D., Laufer I. (2022). EEG pattern classification of picking and coordination using anonymous random walks. Algorithms.

[B102-behavsci-15-00427] Zuckerman I., Mizrahi D., Laufer I. (2023). Attachment style, emotional feedback, and neural processing: Investigating the influence of attachment on the P200 and P400 components of event-related potentials. Frontiers in Human Neuroscience.

